# Aromatase inhibition plus/minus Src inhibitor saracatinib (AZD0530) in advanced breast cancer therapy (ARISTACAT): a randomised phase II study

**DOI:** 10.1007/s10549-023-06873-8

**Published:** 2023-03-02

**Authors:** Ailsa J. Oswald, Stefan N. Symeonides, Duncan Wheatley, Stephen Chan, Adrian Murray Brunt, Karen McAdam, Peter Schmid, Simon Waters, Christopher Poole, Chris Twelves, Timothy Perren, John Bartlett, Tammy Piper, Eve Macdonald Chisholm, Michelle Welsh, Robert Hill, Lisa E. M. Hopcroft, Peter Barrett-Lee, David A. Cameron

**Affiliations:** 1 University of Edinburgh, Edinburgh, Scotland, UK; 2 Royal Cornwall Hospital, Truro, Cornwall England, UK; 3 Nottingham University Hospitals NHS Trust, Nottingham, England, UK; 4 University Hospitals of North Midlands NHS Trust, Stoke-On-Trent & University of Keele, Staffordshire, England, UK; 5 Peterborough City Hospital, Peterborough, England, UK; 6 Barts Cancer Institute, London, England, UK; 7 Velindre Hospital, Whitchurch, Cardiff Wales, UK; 8 University Hospitals Coventry & Warwickshire, Coventry, England, UK; 9 University of Leeds and St James’ Hospital, Leeds, England, UK; 10 University of Edinburgh, Edinburgh, Scotland, UK; 11 Scottish Clinical Trials Research Unit, Edinburgh, Scotland, UK; 12 Velindre Cancer Centre, Cardiff, Wales, UK

**Keywords:** Hormone receptor-positive breast cancer, Endocrine resistance, Src, Bone metastasis

## Abstract

**Purpose:**

The development of oestrogen resistance is a major challenge in managing hormone-sensitive metastatic breast cancer. Saracatinib (AZD0530), an oral Src kinase inhibitor, prevents oestrogen resistance in animal models and reduces osteoclast activity. We aimed to evaluate the efficacy of saracatinib addition to aromatase inhibitors (AI) in patients with hormone receptor-positive metastatic breast cancer.

**Methods:**

This phase II multicentre double-blinded randomised trial allocated post-menopausal women to AI with either saracatinib or placebo (1:1 ratio). Patients were stratified into an “AI-sensitive/naïve” group who received anastrozole and “prior-AI” group who received exemestane. Primary endpoint was progression-free survival (PFS). Secondary endpoints included overall survival (OS), objective response rate (ORR) and toxicity.

**Results:**

140 patients were randomised from 20 UK centres to saracatinib/AI (*n* = 69) or placebo/AI (*n* = 71). Saracatinib was not associated with an improved PFS (3.7 months v. 5.6 months placebo/AI) and did not reduce likelihood of bony progression. There was no benefit in OS or ORR. Effects were consistent in “AI-sensitive/naive” and “prior-AI” sub-groups. Saracatinib was well tolerated with dose reductions in 16% and the main side effects were gastrointestinal, hypophosphatemia and rash.

**Conclusion:**

Saracatinib did not improve outcomes in post-menopausal women with metastatic breast cancer. There was no observed beneficial effect on bone metastases.

CRUKE/11/023, ISRCTN23804370.

## Background

Metastatic breast cancer (MBC) is oestrogen receptor (ER) positive in around 75% of cases [1, 2]. The disease control rate of endocrine therapy in ER-positive MBC is variable, but between 40 and 70% [3]. However, most patients with metastatic disease will develop endocrine resistance, resulting in disease progression and premature death [3]. Though there have been recent advances in prolonging endocrine sensitivity, for example with CDK4/6 inhibitors, endocrine resistance remains a major clinical issue.

Multiple mechanisms of endocrine resistance exist, but one pathway may be through Src activation. Src is a non-receptor tyrosine kinase which is involved in multiple oncogenic pathways with implications for disease activity and therapy resistance [4]. Src activation occurs in up to 40% of oestrogen receptor (ER)-positive breast cancers and has been strongly implicated in endocrine resistance [5–7]. This mechanism is likely via accelerated proteolysis of p27, a mediator of endocrine therapy-induced cell cycle arrest [5, 6]. An increased predisposition towards developing bone metastasis has been associated with Src activity in animal models [8]. Clinically, increased c-Src levels are also associated with a reduction in recurrence-free survival [9].

Saracatinib (AZD0530) is a potent and selective oral inhibitor of Src kinase. It enhances the anti-proliferative effect of endocrine agents in breast cancer models, thereby preventing endocrine resistance development and restoring sensitivity of resistant models to oestrogen deprivation [10]. Pre-clinical data demonstrated that saracatinib could enhance anti-proliferative effects of multiple endocrine agents on breast cancer cell lines [11], with similar results in xenografts [7]. Saracatinib is also known to inhibit bone resorption via osteoclast activity in patients with advanced malignancy (with bone being a common site of metastasis in hormone-sensitive breast cancer) [12, 13].

Phase I trials indicate that saracatinib is well tolerated with mainly gastrointestinal adverse events. Drug half-life is ~ 40–45 h and it is 90% protein bound in plasma with a large volume of distribution [14, 15]. Tumour Src activity is inhibited at doses from 50 mg and above, with the maximum tolerated once daily dose of 175 mg for a European population. [14]

This study was designed to test the hypothesis that the addition of a potent Src inhibitor (AZD0530) to conventional aromatase inhibition would improve outcomes in post-menopausal women with advanced incurable breast cancer. Based on the molecular basis of AZD0530, the presumed mechanism would be by delaying and/or reversing endocrine resistance.

## Materials & methods

### Study design

We conducted a phase II double-blind randomised multicentre study from 2012 to 2015. Trial participants were randomised to receive an aromatase inhibitor (AI) plus either saracatinib (AZD0530) or matching placebo tablets. The saracatinib dose was 175 mg orally once daily, administered with or without food. Participants were enrolled into one of two strata. These were either (i) “AI-sensitive/naïve” who were women deemed to have potentially AI-sensitive tumours, or (ii) “prior-AI” who were a group of women whose cancers had already progressed on an AI, but for whom the treating clinician felt there was likely still some endocrine sensitivity (full eligibility outlined below). The “AI-sensitive/naïve” group were appropriate for a non-steroidal AI and received anastrozole 1 mg daily, plus either saracatinib (AZD0530) or placebo. The “prior-AI” group were appropriate for treatment with a steroidal AI and received exemestane 25 mg daily, plus either saracatinib (AZD0530) or placebo. Of note, selective oestrogen receptor downregulators, such as fulvestrant, were not routinely available at the time this trial was conducted.

There were twenty registering centres. Randomisation of patients to a treatment group (1:1 allocation) was via a central telephone system at the SCTRU (Scottish Clinical Trials Research Unit, Public Health Scotland) clinical trials unit in Edinburgh, a partner in CaCTUS (Cancer Clinical Trials Unit Scotland). Treatment group was allocated using a minimisation algorithm including the following factors: AI sensitivity, disease site (bone metastasis alone versus any other site), concurrent bisphosphonate use, performance status and treatment centre.

Primary analysis was planned after 110 PFS events occurred, or a minimum of 6 months of follow-up in all patients. The study was registered (ISRCTN23804370), sponsored by the Common Services Agency for the Scottish Health Service (UK), partially funded by AstraZeneca (UK) with infrastructure support from the R&D departments of the NHS in the 4 UK nations (NIHR, CSO, HCRW, HSC PHA), and endorsed by Cancer Research UK’s Clinical Trials Awards & Advisory Committee (CTAAC). It was conducted in accordance with ICH Good Clinical Practice and UK National Research Ethics Committee approval was obtained from the West of Scotland Research Ethics Service.

### Eligibility & exclusion criteria

Eligible participants were women with advanced breast cancer suitable for 1st or 2nd line of hormonal treatment. Main eligibility criteria included post-menopausal state, ER-positive disease (Allred score ≥ 3), incurable metastatic disease with at least one measurable lesion, performance status 0–2, life expectancy of > 3 months, HER2 negative (by IHC and/or FISH) or HER2 positive but not a candidate for anti-HER2 therapy, biopsy-confirmed ER-positive disease if bone-only disease and satisfactory haematology/biochemistry results.

Patients also had to meet inclusion criteria for one of the two strata of either “AI-sensitive/naïve” or “prior-AI”. In the “AI-sensitive/naïve” group, patients either had never had an AI (but were permitted to have had prior tamoxifen without rapid progression, defined as having had ≥ 24 months of treatment in the adjuvant setting or ≥ 6 months treatment in the metastatic setting), or had received AI in the adjuvant/neoadjuvant setting (with no progression for at least 12 months whilst not being an on AI). In the “prior-AI” group, they had previously been treated with a non-steroidal AI without rapid progression (that is, for at least 24 months in neoadjuvant/adjuvant setting or 6 months for advanced disease). Patients who had two prior lines of AI therapy were ineligible, unless they switched from one AI to another only due to toxicity in the neoadjuvant/adjuvant setting in the absence of any progression/relapse. Prior chemotherapy in the metastatic setting was allowed and a history of palliative radiotherapy within 4 weeks of trial entry was allowed (provided ≤ 20% of bone marrow was irradiated and there was at least one other progressive measurable bone lesion).

Exclusion criteria included significant co-morbidity, interstitial lung disease, rapidly progressive visceral disease, QTc prolongation, CYP3A4 interactions, contraindication to AZD0530 or AI, and pregnancy/lactation. Concomitant chemotherapy and anti-HER2 therapy were not allowed. However, patients receiving bisphosphonates were eligible, provided these were commenced before, or at, trial entry.

### Baseline assessment, follow-up & monitoring

At baseline, patients had a clinical assessment, radiological assessment and plasma sampling. As part of a translational sub-study, plasma samples were obtained and banked for future biomarker research, with an optional tumour biopsy at baseline and at week 6. Assessments were performed at week 12, week 24 and then 3 monthly. After 18 months, assessments were 6 monthly. Assessments included clinical examination, compliance evaluation, laboratory determinations and tumour assessment by CT scan chest/abdomen/pelvis (plus other imaging/clinical measurements appropriate to site of disease) to monitor measurable lesions using RECIST 1.1 criteria. [16] Toxicities were graded using CTCAE version 4 [17], with guidance on treatment interruption and dose reduction. Treatment continued until one of the following occurred: progression of disease, toxicity (requiring either dose reduction or termination of study drug) or patient choice.

Patients were followed up whilst on therapy for efficacy & toxicity, until either progression or 30 days after cessation of trial therapy (whichever was later). Thereafter, patients were tracked for overall survival only.

### Primary & secondary endpoints

The primary endpoint was investigator-determined progression-free survival (PFS). Secondary endpoints were overall survival (OS), toxicity, objective response rate (ORR) as defined by RECIST 1.1 criteria and best percentage change in RECIST 1.1 measurement (waterfall plot).

### Statistical design

Sample size was calculated based on a hypothesised 50% improvement in PFS, with 80% power at a 10% 1-sided level of statistical significance (or 90% power at a 20% level of statistical significance), which would require 140 patients to be recruited over 3 years. A hierarchical approach to progression (or not) to a separate phase III study was planned: (i) if there was an observed difference in favour of AZD0530 at the 10% level then this would be an indication to initiate a subsequent phase III study, (ii) if a favourable result was observed at 20% level (but not 10%) then proceeding to phase III trial would only occur if review of the best percentage change in RECIST 1.1 measurement (waterfall plot) supported clinically meaningful activity or (iii) if there was not a statistically significant benefit at 20% level then it would not be worthwhile proceeding to a phase III trial. [18]

Analysis was conducted on an intention-to-treat basis. Survival analysis was performed using the cox proportion hazard model, using a 1-sided p value. Response rates were compared using Pearson’s chi-square test. Toxicity grading analysis was conducted using the Mann–Whitney U test. Data analysis was performed using SPSS and R (version 3.5.1).

## Results

### Baseline characteristics

**Fig. 1:**
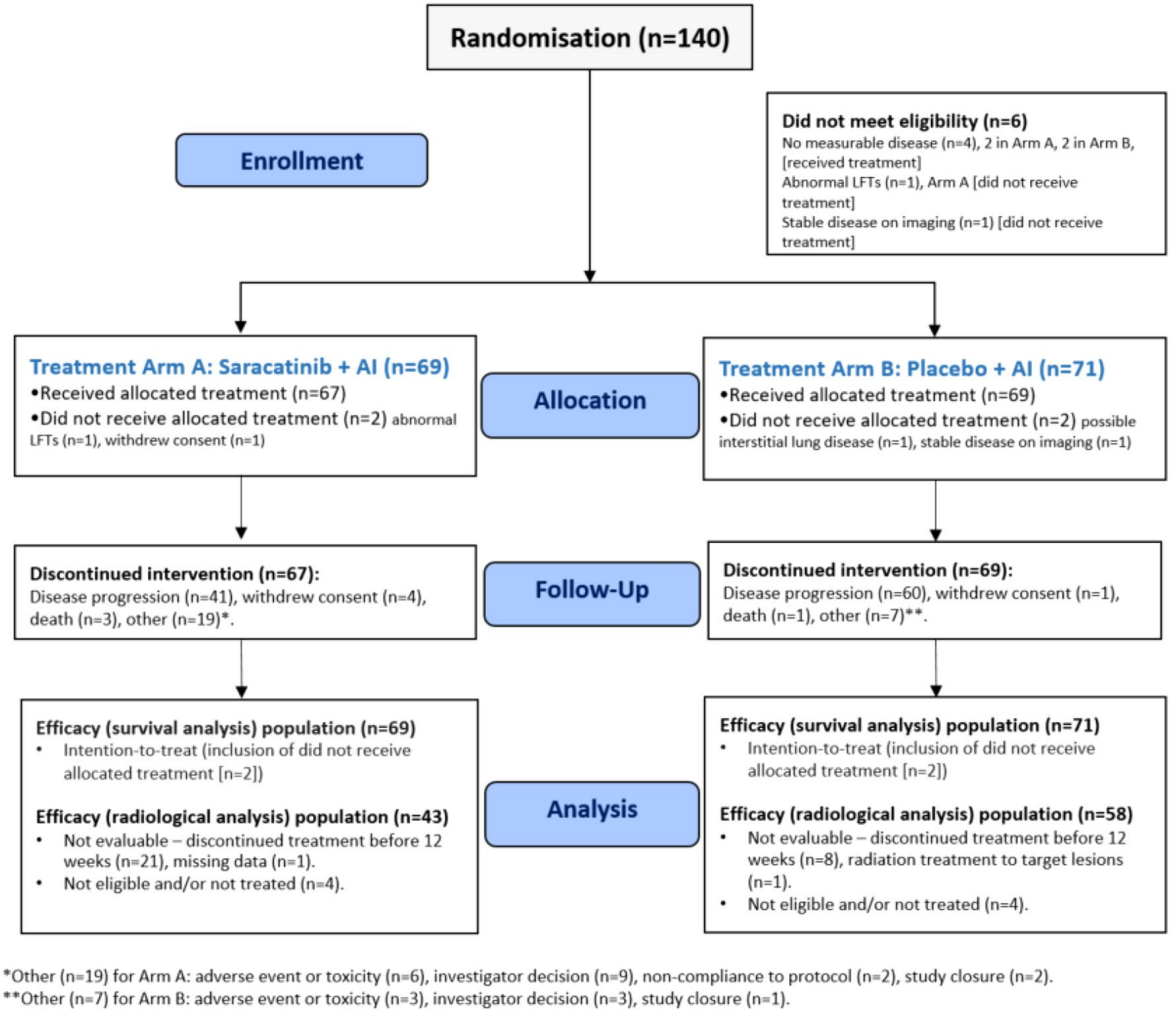
Trial profile

The trial consort diagram is shown in Fig. 1. Between August 2012 and April 2015, 140 patients were enrolled. Four patients were not treated (two in each arm) and therefore 136 (97.1%) patients were included in the safety analysis. Six patients were found to be ineligible after randomisation.

Patient and tumour characteristics are illustrated in Table 1. Both groups were well matched in terms of age, AI sensitivity, histology and previous treatments. Few patients had bone-only disease (*n* = 2 [3%] saracatinib/AI, *n* = 4 [6%] placebo/AI) and around half of patients were on bisphosphonates (*n* = 37 [54%] saracatinib/AI, *n* = 37 [47%] placebo/AI). A small proportion (*n* = 8 [12%] saracatinib/AI, *n* = 11 [16%] placebo/AI)) had previously received palliative chemotherapy. No patients had previously received everolimus or CDK4/6 inhibitors as these were not yet in routine use at the time of enrolment.

**Table 1:** Baseline characteristics of trial population

		Saracatinib/AI	Placebo/AI
		(*n* = 69)	(*n* = 71)
Age [years]		64 [41–88]	65 [41–80]
Aromatase inhibitor (AI) Sensitivity	Sensitive/Naive	36 (52.2%)	33 (46.5%)
	Prior	33 (47.8%)	38 (53.5%)
Performance status	0	38 (55.1%)	42 (59.2%)
	1	29 (42.0%)	24 (33.8%)
	2	2 (2.9%)	5 (7.0%)
Primary tumour type	Ductal NST	51 (73.9%)	53 (74.6%)
	Lobular	15 (21.9%)	13 (18.3%)
	Other	8 (11.6%)	9 (12.7%)
HER2 status	Negative	66 (95.7%)	68 (95.8%)
	Positive	3 (4.3%)	2 (2.8%)
	Unknown	0 (0.0%)	1 (1.4%)
Surgery performed at primary diagnosis	Breast-conserving	22 (31.9%)	29 (40.8%)
	Mastectomy	35 (50.7%)	33 (46.5%)
	Axillary	46 (66.7%)	47 (66.2%)
	No surgery	13 (18.8%)	14 (19.7%)
Prior endocrine therapy	Tamoxifen	39 (56.5%)	46 (64.8%)
	Adjuvant or neoadjuvant	37 (53.6%)	42 (59.2%)
	Metastatic	2 (2.9%)	4 (5.6%)
	Anastrozole	22 (31.9%)	27 (38.0%)
	Adjuvant or neoadjuvant	17 (24.6%)	18 (25.4%)
	Metastatic	5 (7.2%)	9 (12.7%)
	Letrozole	21 (30.4%)	23 (32.3%)
	Adjuvant or neoadjuvant	11 (15.9%)	15 (21.1%)
	Metastatic	10 (14.5%)	8 (11.3%)
	Exemestane	4 (5.8%)	3 (4.2%)
	Adjuvant or neoadjuvant	4 (5.8%)	3 (4.2%)
	Metastatic	0 (0.0%)	0 (0.0%)
Prior radiotherapy		55 (79.7%)	50 (70.4%)
Previous chemotherapy	Any indication	37 (53.6%)	33 (46.4%)
	Neoadjuvant	9 (13.0%)	9 (12.7%)
	Adjuvant	27 (39.1%)	20 (28.2%)
	Palliative	8 (11.6%)	11 (15.5%)
	1 line	6 (8.7%)	5 (7.0%)
	2 or more lines	2 (2.9%)	6 (8.5%)
Disease site (metastasis)	Bone only	2 (2.9%)	4 (5.6%)
	Other	67 (97.1%)	65 (91.5%)
Bisphosphonate use		37 (53.6%)	37 (47.1%)

### Progression-free survival (PFS)

**Fig. 2:**
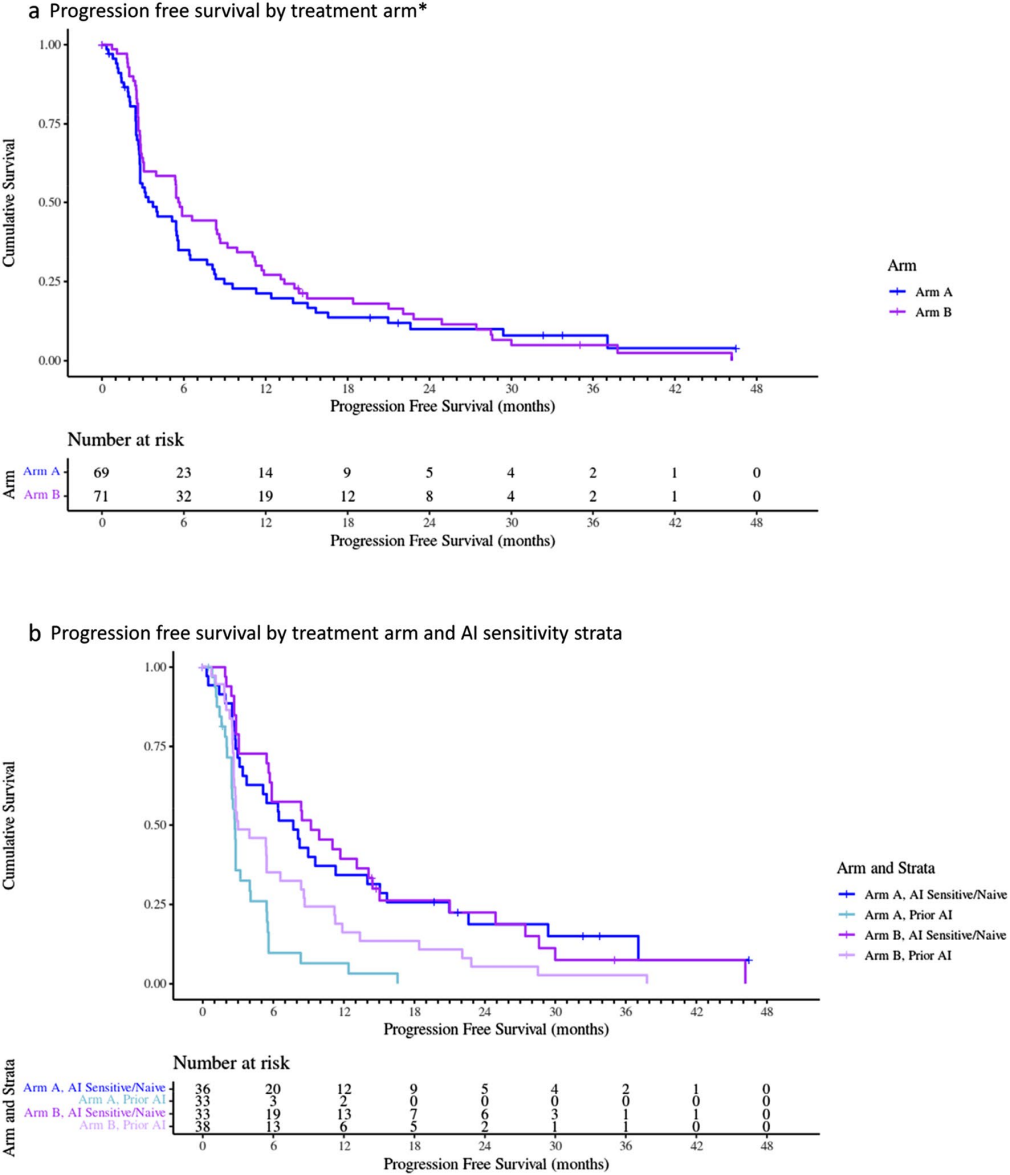
Progression-free survival in treatment arms (**a**) and by AI-sensitivity strata (**b**) *Censored data for PFS by treatment arm: Arm A censored *n* = 8 (11.6%), Arm B censored *n* = 4 (5.6%). Reasons for censoring included withdrew consent (Arm A *n* = 2, Arm B *n* = 0), withdrew due to investigator decision (Arm A *n* = 3, Arm B *n* = 1), withdrew for other reason (Arm A *n* = 0, Arm B *n* = 1), ineligible (Arm A *n* = 1, Arm B *n* = 1), study closure (Arm A *n* = 2, Arm B *n* = 0), missing data (Arm A *n* = 0, Arm B *n* = 1)

The median follow-up for PFS was 10.2 months for the saracatinib/AI group (IQR 4.8–21.8 months) and 16.0 months (IQR 9.4–24.9 months) for the placebo/AI group (Fig. 2a). In the saracatinib/AI arm, the PFS was 3.7 months ([95% CI 1.4–6.0], 61 events), compared with 5.6 months in the placebo/AI group ([95% CI 4.4–6.8], 67 events, one sided *p* = 0.99)*.* There was no evidence to suggest the addition of saracatinib resulted in an improved PFS. Data on PFS were similar between treatment arms when comparing those in the “AI-sensitive/naïve” subgroup (saracatinib/AI 7.7 months [95% CI 4.5–10.9], placebo/AI 9.2 months [95% CI 3.4–15.0]) and the “prior-AI” subgroup (saracatinib/AI 2.7 months [95% CI 2.5–2.9], placebo/AI 3.0 months [95% CI 0.4–5.6]), as shown in Fig. 2b.

### Overall survival (OS), objective response rate (ORR), tumour size & sites of progression

In the saracatinib/AI group, OS was 24.1 months [95% CI 17.0–31.1], compared with 22.9 months [95% CI 19.5–26.3] in the placebo/AI group (one sided *p* = 0.88), indicating no significant difference in OS between treatments arms. When conducting a planned subgroup analysis, OS data were similar in the “AI-sensitive/naïve” (saracatinib/AI 24.6 months [95% CI 17.0–32.2], placebo/AI 32.0 months [95% CI 24.2–39.8]) and the “prior-AI” subgroup (saracatinib/AI 17.6 months [95% CI 8.2–27.1], placebo/AI 17.3 months [95% CI 15.4–19.2]).

The total number of deaths in the saracatinib/AI group was 39 (55%). Thirty-four (87%) of those were related to breast cancer and 5 (13%) were unrelated (infection/sepsis [*n* = 2], pulmonary emboli [*n* = 2], unknown [*n* = 1]). In the placebo/AI group, there were 41 (58%) deaths, with 39 (95%) of those related to breast cancer and 2 (5%) unrelated deaths (infection/sepsis [*n* = 1], dementia [*n* = 1]).

**Fig. 3:**
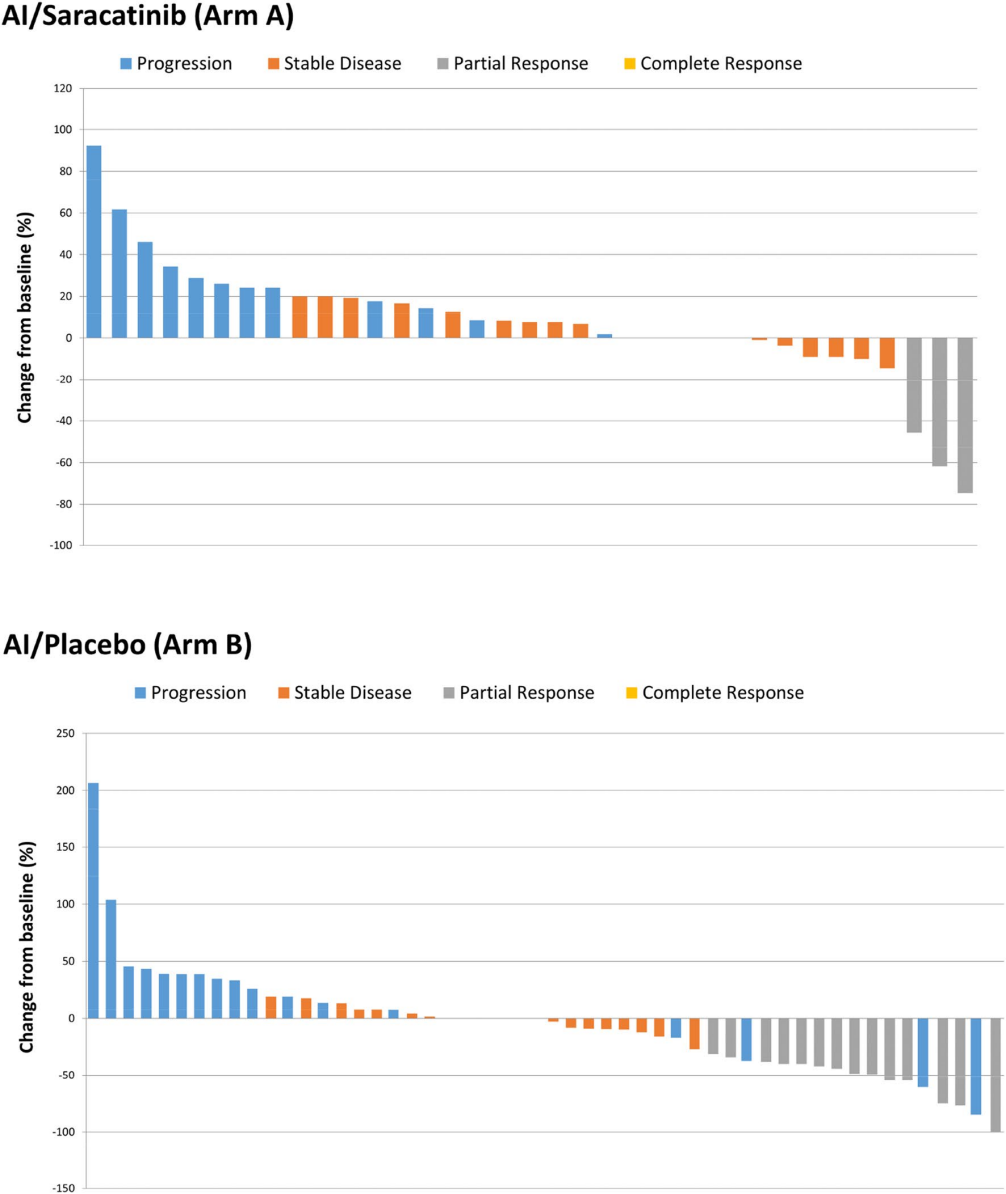
Overall response rate comparing saracatinib/AI and placebo/AI

Objective response rate is illustrated in Table 2, with waterfall plot analysis in Fig. 3. There was a similar proportion of patients (saracatinib/AI vs. placebo/AI) with progressive disease (23% vs. 25%) and stable disease (30% vs. 31%) as best response in each arm*.* There was a numerically higher rate of response (partial or complete) in those treated with placebo/AI (27%) compared to saracatinib/AI (8%). There was also no significant difference in mean change of tumour diameter when comparing groups (+ 56% saracatinib/AI vs. + 44% placebo/AI, *p* = 0.48).

**Table 2:** Overall Response Rate comparing Saracatinib/AI and Placebo/AI

	Saracatinib/AI (*n* = 65)*	Placebo/AI (*n* = 67)*
Complete response	1(1.5%)	1 (1.5%)
Partial response	4 (6.2%)	17 (25.4%)
Stable disease	20 (30.8%)	21 (31.3%)
Progressive disease	15 (23.1%)	17 (25.4%)
Not evaluable for response	25 (38.5%)	11 (16.4%)
Progression prior to first trial assessment**	11 (16.9%)	7 (10.5%)
Other***	14 (21.5%)	4 (6.0%)

^*^Note that those ineligible and/or untreated were excluded. (Arm A [saracatinib/AI] *n* = 4, Arm B [placebo/AI] *n* = 4)

^**^Although not formally evaluable, these patients stopped due to progression prior to first trial assessment at 12 weeks (either clinical progression or progression on non-trial imaging)

^***^Other reasons for being non-evaluable**. **Arm A [saracatinib/AI]: Investigator decision (*n* = 5), patient choice/withdrawal of consent (*n* = 5), drug toxicity (*n* = 3), death (*n* = 1). Arm B [placebo/AI]: investigator decision (*n* = 1), patient choice/withdrawal of consent (*n* = 1), drug toxicity (*n* = 1), radiation to target lesion (*n* = 1). [Note any other explanation was prioritised over “investigator decision”]

There was a sizeable proportion of the study population that were not evaluable for response rate and this was also imbalanced between treatment arms (saracatinib/AI 39%, placebo/AI 14%). In the majority of cases, this was because patients did not reach their first assessment imaging at 12 weeks due to disease progression. Other reasons included investigator decision, withdrawal of consent or toxicity, as detailed in Table 2. Given the imbalance across the two arms, a sensitivity analysis of all efficacy endpoints was conducted in the subpopulation of those who reached their 12 week scan. Results were similar in this subgroup (saracatinib/AI n = 40 [58%], placebo/AI n = 58 [82%]) to those seen in the wider population, with no difference in PFS (5.5 months, vs. 6.6 months, *p* = 0.31) or OS (24.8 months, vs. 24.1 months, *p* = 0.50).

In terms of progressive disease site (saracatinib/AI vs. placebo/AI), a similar proportion progressed in their existing site (42% vs. 41%) compared with new site only (19% vs. 18%). There was a numerically higher proportion of those in placebo/AI group (32%) progressing in both existing and new disease site compared to the saracatinib/AI group (16%). Commonest disease site location for progression (saracatinib/AI vs. placebo/AI) was liver (19% vs. 23%), bone (9% vs. 6%), lymph nodes (3% vs. 15%) or lungs (3% vs. 10%).

New bone metastases were observed at least as often with saracatinib/AI (*n* = 5) as with placebo/AI (*n* = 3). However, for those patients with bone-only disease at enrolment (saracatinib/AI *n* = 2, placebo/AI *n* = 4) the only patient that did not have bony progression was in the saracatinib/AI arm.

### Toxicity & dose reductions

Dose interruptions and reductions were relatively uncommon. In both arms, patients received a median of 100% of the intended doses of saracatinib or placebo whilst on treatment (mean of 95% doses in saracatinib/AI arm, 99% placebo/AI arm). Nineteen percent in the saracatinib/AI group required a first dose reduction (to 125 mg) compared to 10% in placebo/AI group. A further two patients (3%) had a second dose reduction (to 50 mg) in the saracatinib/AI group, with no further reductions in the placebo/AI group. The most common reasons for dose reduction (saracatinib/AI vs. placebo/AI) were gastrointestinal side effects (8% vs. 3%), rash (3.0% vs. 0%) and fatigue (3.0% vs. 1%).

Grade 1–4 adverse events in each treatment arm are reported in Table 3. The most common toxicity in both groups was fatigue (74.6% saracatinib/AI vs. 65.2% placebo/AI) with no significant difference between groups. There was a significantly higher proportion of patients reporting the following adverse events in the saracatinib/AI group compared with placebo/AI group: hypophosphatemia (*p* < 0.001), anorexia (*p* = 0.004), vomiting (*p* = 0.02), alopecia (*p* = 0.02) and rash (*p* = 0.04).

**Table 3:** Comparison of Toxicities in Saracatinib/AI and Placebo/AI

	Saracatinib/AI *n* = 67		Placebo/AI *n* = 69		
Toxicity	Grade 1/2 *n* (%)	Grade 3/4 *n* (%)	Grade 1/2 *n* (%)	Grade 3/4 *n* (%)	
Fatigue	46 (68.7%)	4 (6.0%)	44 (63.8%)	1 (1.5%)	*p* = 0.09
Alopecia	17 (25.4%)	0 (0.0%)	4 (5.8%)	0 (0.0%)	*p* = 0.02
Vomiting	19 (28.3%)	3 (4.5%)	10 (14.5%)	0 (0.0%)	*p* = 0.02
Anorexia	29 (43.3%)	0 (0.0%)	13 (18.8%)	0 (0.0%)	*p* = 0.004
Diarrhoea	20 (30.0%)	1 (1.5%)	11 (15.9%)	0 (0.0%)	*p* = 0.05
Rash	22 (32.8%)	1 (1.5%)	8 (11.6%)	0 (0.0%)	*p* = 0.04
Infections	17 (25.4%)	5 (7.5%)	27 (39.1%)	4 (5.8%)	*p* = 0.28
Low phosphate	19 (28.4%)	8 (12.0%)	4 (5.8%)	0 (0.0%)	*p* < 0.0001
Low potassium	7 (10.5%)	0 (0.0%)	2 (2.9%)	0 (0.0%)	*p* = 0.07

### Factors predicting better outcome

As expected, those who were “AI-sensitive/naïve” had a better survival outcome compared with those in the “prior-AI group”, with a similar effect in both treatment arms, as described above and seen in Fig. 2b. Analysis was also adjusted for other variables including disease site and performance status, which did not alter outcome. Bisphosphonate use was associated with an increased PFS (HR 0.57, 80% CI 0.45–0.73, *p* = 0.004) and increased OS (HR 0.48 80% CI 0.35–0.66, *p* = 0.003). Patients receiving saracatinib were just as likely to progress with bone metastasis (9%) as those receiving placebo (6%).

## Discussion

This phase II double-blind randomised study was designed to investigate the benefit of the addition of saracatinib, a Src inhibitor, to standard aromatase inhibition in metastatic hormone-sensitive breast cancer. It did not find any evidence of enhanced anti-tumour activity. No statistically significant benefit was observed in the primary endpoint of PFS (3.7 months saracatinib/AI vs. 5.6 months placebo/AI), or in other endpoints, whether patients were receiving their first line of aromatase inhibition, or their second. Numerically, fewer patients continued to 12 weeks of imaging in the saracatinib/AI treatment arm and a lower proportion had a radiological response.

This trial was conducted between 2012 and 2015 and therefore is reflective of clinical practice at that time. Since then, there have been major advances in tackling oestrogen resistance in metastatic breast cancer. Everolimus (an mTOR inhibitor) was approved in combination with exemestane by the European Medicines Agency in 2012 and by the National Institute for Health and Care Excellence (NICE) in 2016. This was following the BOLERO-2 study, which included patients with disease recurrence or progression with a non-steroidal AI [19]. Endocrine therapy in combination with CDK4/6 inhibitors[20, 21] followed closely, with NICE approvals from 2017 onwards. In this study, the median PFS in our “prior-AI group” was comparable to the exemestane/placebo arm of BOLERO-2 (around 3 months) [19]. However, we note the OS in both our treatment arms was poor in comparison to control arms of other clinical trials [22, 23]. This may be reflective of our relatively open inclusion criteria or differences in subsequent treatments. Although our trial population differs from current practice, these results do provide a relevant negative finding for the potential role of saracatinib, and related Src inhibitors, in treating metastatic ER-positive breast cancer. Src remains of interest as a target in breast cancer and, interestingly, one mechanism of CDK4/6 inhibitor resistance may involve downregulation of p27^kip1^, which occurs via phosphorylation of Src [24]. Pre-clinical work demonstrates that cell lines with high levels of phosphorylated p27^kip1^ were resistant to CDK4/6 inhibitor, but co-administration of saracatinib restored CDK4/6 inhibitor sensitivity in vitro and in vivo (albeit in a colorectal cancer model).[25] There is an ongoing phase I study investigating the combination of another Src inhibitor (bosutinib) with a CDK4/6 inhibitor and fulvestrant in advanced breast cancer refractory to a CDK4/6 inhibitor (NCT03854903). However, we believe that the outcome of our own trial is unlikely to have been significantly altered in a current context of prior CDK4/6 or mTOR inhibition.

There are a number of potential reasons for the observed lack of benefit of saracatinib. Importantly, there is evidence that this generation of Src inhibitors inhibit kinase activity but may actually stabilise the active conformation of the protein, resulting in contradictory effects via scaffold interactions [26]. Pharmacological selectivity is another consideration, as c-Abl is also commonly inhibited and this has been shown to promote cell proliferation in vitro.[27] Lack of patient selection may also have contributed, as Src activation is only present in around 40% of ER-positive breast cancers [7]. There have been attempts to identify predictive gene signatures and biomarkers for patient selection for Src inhibitors, but these have unfortunately been unsuccessful [28, 29].

The lack of benefit is unlikely to be explained by dosing issues, given that previous phase I/II trials have suggested a dose of 175 mg saracatinib was adequate to inhibit Src kinase activity [14, 30]. However, we did not repeat pharmacodynamic analysis within this study. Similarly, we did not perform pharmacokinetic analysis, given the prior phase I data [14] had matched pre-clinical data and no potential interaction with AI was anticipated. Hence, it is not possible to definitively exclude this explanation within the current study.

The lack of benefit illustrated from our study is disappointing, particularly in the context of well-established importance of Src in cancer and supportive pre-clinical data [31]. Other clinical trials of Src inhibitors in metastatic breast cancer have also largely been disappointing. A phase II trial of single agent saracatinib in ER-negative metastatic breast cancer demonstrated no efficacy with significant toxicity and terminated early [32]. Other Src inhibitors, such as bosutinib and dasatinib, have demonstrated disappointing results in patients with hormone receptor-positive metastatic breast cancer. A phase II study of bosutinib combined with endocrine therapy had to end prematurely due to significant toxicity. [33, 34] The addition of dasatinib to letrozole in a phase II study failed to demonstrate a difference in clinical benefit rate (their primary endpoint) but did slightly improve median PFS [35]. No benefit was noted in other trials which investigated dasatinib with exemestane or fulvestrant. [36, 37] There are no phase III trials with Src inhibitors reported in breast cancer.

Given the link with Src and osteoclast function [13, 38], Src inhibitors have been highlighted as a potential therapeutic target for bone metastasis [39]. Saracatinib was not effective for cancer-induced bone pain in a phase II trial, but it did result in reduced bone resorption [40, 41]. In our study, those treated with saracatinib did not have a lower likelihood of developing new bone metastasis. However, our analysis on bone metastasis could be limited by both the small population with bone-only disease (*n* = 6) and the sensitivity of CT imaging (as opposed to isotope bone scan or PET scan). Interestingly, saracatinib is now being investigated in non-malignant bone conditions such as fibrodysplasia ossificans progressiva (a rare connective tissue disorder characterised by abnormal bone development in soft tissue areas). [42]

Next generation, more selective Src inhibitors are currently being developed and tested in pre-clinical environments. [43, 44] These include the novel Src kinase inhibitor NXP900, with a unique and novel mechanism of Src inhibition by interfering with catalytic and scaffolding functions, by targeting the native inactive conformation of Src. This compound demonstrated higher potency and selectivity than any other Src inhibitor, on a panel of breast cancer cell lines and also demonstrated potency in vivo. [44], [45]

Overall, these data do not support further evaluation of saracatinib in combination with AIs in advanced hormone-sensitive breast cancer. Despite approvals of other drugs, such as CDK4/6 inhibitors, endocrine resistance remains an important target in metastatic breast cancer and novel treatment options are required. The importance of Src in breast cancer is well established but unfortunately targeting Src with this generation of Src inhibitors has not been successful. However, novel more selective Src inhibitors may be a more promising avenue, including in a CDK4/6 inhibitor-resistant population.

## Data Availability

No publicly available datasets.
